# *Rhipicephalus simus* ticks: new hosts for phleboviruses

**DOI:** 10.1017/S0031182024001033

**Published:** 2024-08

**Authors:** Samuel Munalula Munjita, Benjamin Mubemba, John Tembo, Mathew Bates, Sody Munsaka

**Affiliations:** 1 Department of Biomedical Sciences, School of Health Sciences, University of Zambia, Lusaka, Zambia; 2 Department of Wildlife Sciences, School of Natural Resources, Copperbelt University, Kitwe, Zambia; 3 HerpeZ, University Teaching Hospital, Lusaka, Zambia; 4 School of Life Sciences, University of Lincoln, Lincoln, Lincolnshire, UK

**Keywords:** Ixodid tick, mNGS, novel, phlebovirus, *Rhipicephalus simus*

## Abstract

Ticks are widespread arthropods that transmit microorganisms of veterinary and medical significance to vertebrates, including humans. *Rhipicephalus simus*, an ixodid tick frequently infesting and feeding on humans, may play a crucial role in transmitting infectious agents across species. Despite the known association of many *Rhipicephalus* ticks with phleboviruses, information on *R. simus* is lacking. During a study in a riverine area in Lusaka Zambia, ten *R. simus* ticks were incidentally collected from the grass and bushes and subjected to metagenomic next generation sequencing (mNGS) in 2 pools of 5. Analysis detected a diverse microbial profile, including bacteria 82% (32/39), fungi 15.4% (6/39), and viruses 2.6% (1/39). Notably, viral sequence LSK-ZM-102022 exhibited similarity to tick phleboviruses, sharing 74.92% nucleotide identity in the RdRp gene and 72% in the NP gene with tick-borne phlebovirus (TBPV) from Greece and Romania, respectively. Its RNA-dependent RNA polymerase (RdRp) encoding region carried conserved RdRp and endonuclease domains characteristic of phenuiviridae viruses. Phylogenetic analysis positioned LSK-ZM-102022 in a distinct but lone lineage within tick phleboviruses basal to known species like brown dog tick phlebovirus and phlebovirus Antigone. Pair-wise genetic distance analysis revealed similar findings. This study emphasizes the urgency of further research on the ecology, transmission dynamics, and pathogenic potential of LSK-ZM-102022 and related TBPVs, crucial for local and global preparedness against emerging tick-borne diseases.

## Graphical abstract

**Figure:**
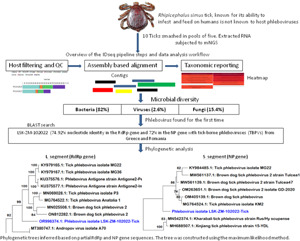

## Introduction

Ticks are widespread blood feeding arthropod ectoparasites of mammals, reptiles and other vertebrate animals (Bonnet *et al*., 2017). Among these, *Rhipicephalus simus*, classified within the genus *Rhipicephalus* and the family Ixodidae, stands out as a highly capable vector of pathogens that hold critical importance in both medical and veterinary fields (Xu *et al*., 2016; Shekede *et al*., 2021; Phiri *et al*., 2023). This hard tick species not only thrives in diverse habitats but also exhibits a remarkable ability to infest and feed on humans, thus potentially facilitating the transmission of a wide range of infectious agents (Horak *et al*., 2002). Adult *R. simus* ticks largely prefer monogastric animals, equids and suids as their hosts (Horak *et al*., 2002). Distinctive odours associated with monogastric diets and digestive processes are speculated to play a pivotal role in attracting *R. simus* ticks to humans and other monogastric animals (Horak *et al*., 2002). The tick under consideration a known vector for the zoonotic pathogen, *Rickettsia conorii* (Beati *et al*., 1995). Other pathogens detected in *R. simus* so far include *Ehrlichia minasensis*, *Theiliera* sp., *Babesia caballi*, *Hepatozoon* sp*., Anaplasama* sp., *Rickettsia massiliae*, and Candidatus *Rickettsia barbariae* (Parola, 2006; Ledger *et al*., 2021). Viruses have scarcely been reported in *R. simus* ticks despite increasing reports of tick-borne viruses globally (Haig *et al*., 1965; Burt *et al*., 1996; Bartíková *et al*., 2017).

Phleboviruses belonging to the genus *Phlebovirus* and family *Phenuiviridae,* frequently identified in ticks of the genus *Rhipicephalus* worldwide (Li *et al*., 2016; Pereira *et al*., 2017; López *et al*., 2020; Simulundu *et al*., 2021), have not been reported in *R. simus*. Actually, in sub-Saharan Africa, there are limited reports of phleboviruses in tick species except those involving *Rhipicephalus appendiculatus*, *Amblyomma* sp., and *Hyalomma* sp (Kobayashi *et al*., 2017; Simulundu *et al*., 2021; Amoa-Bosompem *et al*., 2022; Ogola *et al*., 2023). Tick-borne phleboviruses (TBPVs) were largely neglected until recently when severe fever with thrombocytopenia syndrome virus (SFTSV) and Heartland virus (HRTV) were confirmed as causative agents of severe disease in humans (Li *et al*., 2016; McMullan Laura et al., 2012). The genus *Phlebovirus* has about 82 officially classified viruses whose genomes are negative sense single-stranded RNAs divided into three segments (L, M and S) (Abudurexiti *et al*., 2019; ICTV, 2024). Segment L encodes the RNA-dependent RNA polymerase (RdRp), segment M encodes the viral glycoproteins (GP), while S segment encodes the nucleocapsid protein (NP) (Abudurexiti *et al*., 2019; ICTV, 2024).

Given the substantial emerging risk posed by TBPVs, a metagenomic next-generation sequencing (mNGS) study was conducted to determine the viral diversity within tick populations from a dormant commercial farm in a riverine area in Lusaka, Zambia. The findings mark the first identification of a phlebovirus in *R. simus* ticks anywhere in the world. The report underscores the critical need for intensified surveillance and comprehensive research on tick-borne viruses in Zambia and the broader sub-region.

## Materials and methods

### Study area

The study was an incidental offshoot of a larger study focused on mammarenaviruses in rodents conducted partly in a riverine area on the outskirts of Lusaka city, the capital of Zambia (Munjita *et al*., 2022). Therefore the study area, as previously reported (Munjita *et al*., 2022), was a dormant commercial farm (S15°26′16.511″, E 28°26′22.174″) bordered by two seasonal streams. Ecologically, the study site was partially disturbed with on and off farming activities. The area had a dense cover of grass (*Cenchrus purpureus and Cynodon nlemfuensis*). Acacia trees were the common tree types.

### Tick collection

In March 2022, 10 ticks were incidentally collected from the grass and bushes on a commercial farm in Lusaka, Zambia. Ticks collected from the grasses were separated from those from the bushes. The month of March falls in the midst of the rainy season in Zambia, a period characterised by increased vegetation growth and heightened activity of various arthropods, including ticks (World Bank, 2021). The collected ticks were stored in 1.8 mL flat-bottomed, screw-capped tubes, each containing a green leaf to maintain moisture levels. This precautionary measure ensured the ticks remained viable until laboratory processing. The samples were transported to the laboratory in a portable cool box packed with ice packs to maintain a low temperature and preserve the integrity of the specimens. Upon arrival at the laboratory, the ticks underwent microscopical morphological identification to determine their species. Thereafter, the 10 ticks were pooled into 2 groups of 5 ticks each based on the location from which they were found (bushes – pool 1 or grass – pool 2). Following identification and pooling, the samples were stored at −30°C for 24 hours to ensure they remained viable for subsequent analyses. The next day, nucleic acid (RNA) extraction was performed on the preserved pooled tick samples.

### Identification of ticks

The ticks were identified morphologically under a light microscope Leica S9E (Leica Microsystems, Heerbrugg, Switzerland) with the help of a reference book (Walker *et al*., 2000; Walker and Bouattour, 2003). The identity of the ticks was confirmed using available mNGS generated host small subunit ribosomal RNA (ssrRNA) and internal transcribed spacer (ITS) region assembled nucleotide sequences. Two sets of genetic sequences, each spanning 252 bp ssrRNA and 653 bp ITS, were employed to verify the species of ticks present in the first pool, while 2 additional ssrRNA sequences, totalling 1595 bp and 309 bp, were utilized to confirm the species of ticks in the subsequent pool. The use of rRNA and ITS to identify ticks has been reported before (Barker, 1998; Fukunaga *et al*., 2000; Intirach *et al*., 2023; Kim *et al*., 2024).

### RNA extraction

The 2 pools of ticks were homogenized in Phosphate-buffered saline (PBS) in separate 1.8 mL flat bottomed screw capped tubes. Total RNA was extracted from the supernatant of the homogenized ticks using the QIAamp Viral RNA Mini Kit (Qiagen, Hilden, Germany) according to the manufacturer's instructions. Extracted total RNA was mixed with equal volumes of RNA later (ThermoFisher Scientific, Waltham, MA, USA) and stored at −80°C for 5 days until samples were transported to the USA for mNGS.

### Library construction and sequencing

RNA samples from the ticks were subjected to mNGS at the Chan-Zuckerberg (CZ) Biohub, San Francisco, United States of America (USA). The library preparation protocol was adapted from the NEBNext Ultra II RNA protocol (New England Biolabs, Ipswich, MA, USA) with minor modifications as previously reported (Munjita *et al*., 2022). Briefly, RNA was fragmented and spiked with External RNA Controls 103 Consortium collection (ERCC) (ThermoFisher, Waltham, MA, USA). The spiked RNA was converted into complementary DNA (cDNA) followed by ligation with adaptors and then digestion of adapters, barcoding, and final clean-up on a magnetic rack. The 4150 Tapestation system (Agilent, MA, USA) was used to determine the size and concentration of the library. The flow cell containing the library was washed to remove salts followed by denaturing of the library with sodium hydroxide (NaOH) (0.2 N). PhiX was added as a calibration control for the Illumina sequencing platform. Thereafter, the sample libraries were loaded onto the Illumina Novaseq 6000 (Illumina, 97 San Diego, CA, USA) sequencer.

### Analysis of mNGS data and quality control metrics

Raw mNGS data was analysed in the CZ ID sequencing pipeline (IDseq) portal. IDseq is an open-source cloud-based bioinformatics platform (Kalantar *et al*., 2020). The analysis of generated sequence reads included 3 steps: host and quality filtration, alignment based assembly and assignment of reads and contigs to taxa, and finally reporting and visualization which included application of a background model. Briefly, if a read was incorporated into a contig, it was given the taxonomic identifier belonging to the National Centre for Biotechnology Information (NCBI) accession number to which its parent contig was assigned. Reads for each sample were subjected to quality control checks including a set of filters, which required nucleotide reads per million sequenced to be 500 or more (NT_rPM ≥ 500), average taxon length of 200 bp and *Z* score equal to one (*Z* Score = 1). The *Z*-score statistic was computed due to the application of a background model to remove taxa that may have been prevalent in water controls and passed through filtration. The background correction model was derived from negative water controls. Following application of these filters, a consensus genome for a particular viral infectious agent was generated and downloaded. Only available viral gene segments were included in the analysis. For other pathogen types (bacteria, fungi and others), the pipeline did not provide options to create a consensus genome, thus, individual contigs of 200 bp or more aligning to a specific section of the genome were downloaded for further analysis such as Basic Local Alignment Search Tool (BLAST) search. With regards to infectious disease agent load, the pipeline supported the determination of the relative abundance of particular taxa based on reads aligning to sequences in the NCBI.

### BLAST search, conserved domain search, phylogenetic analysis, and pair-wise distances

BLAST search was performed using the query sequences against the NCBI nucleotide sequences. The search parameters were left on default. The results were analysed for significant matches, and some of the sequences formed part of the data set for phylogenetic analysis. Conserved domains (CD) in protein sequences were identified through CD-Search tool in BLAST. Phylogenetic analysis was performed using MEGA11 software (Tamura *et al*., 2021), employing the Maximum Likelihood method with the General Time Reversible model for nucleotide sequences and the LG + G model for amino acid sequences (Kimura, 1980). The reliability of the phylogenetic tree was assessed using the bootstrap method with 1000 replicates. Prior to the phylogenetic analysis, the data set consisting of aligned sequences underwent a model selection test within MEGA11. This was accomplished by accessing the models tab and evaluating available options. A model with the lowest Bayesian Information Criterion (BIC) score was selected, as it was deemed the most appropriate for accurately describing the evolutionary substitution pattern. To understand the degree of genetic variation between the sequences from this study and related organisms, pair-wise distances were determined in MEGA 11 using aligned sequences.

## Results

### Identities of the collected ticks

The number of mNGS reads aligning to *R. simus* in the NCBI database were 4, 811 higher in pool 1 than pool 2 (Table 1). However, pool 1 contained numerous contigs that were shorter in length, with an average contig size of 260 base pairs (bp), in contrast to pool 2's average contig size of 852 bp. The total number of reads across all assembled contigs for *R. simus* was almost 2-fold higher in pool 2 than pool 1. With regards to relative abundance of nucleic acid associated with each *R. simus* taxon in the sample, pool 2 from the bushes had high reads than pool 1 from the grass. Based on mNGS data and initial microscopic analysis, ticks were identified to be *R. simus*. The sequences were deposited in the GenBank database under accession numbers PP355687 (ssRNA, mitochondrial) and PP357248 (ITS) for the first pool, as well as PP356686 (ssRNA) and PP375807 (ssRNA, mitochondrial) for the second pool.

**Table 1.** mNGS metrics for reads and contigs aligning to *R simus*

Metric	Value	
	Pool 1	Pool 2
r (number of reads aligning to taxon in the NCBI NT/Nr database)	95 436	90 485
Total number of reads across all assembled contigs (Contigs r)	49 010	82 811
Number of assembled contigs aligning to *Rhipicephalus simus*	87	18
Average length (L) of the local alignment for all contigs for all contigs and reads assigned to *Rhipicephalus simus*	260 bp	852 bp
Reads per million (number of reads aligning to the taxon in the NCBI NT or NR database, per million reads sequenced)	97 436	140 565

### Microbial diversity

A total of 1,629,728 reads were determined from the 2 pools of ticks but only 26.9% (437 694) remained for analysis after QC filtering and removal of host reads. Further QC filtering to remove low quality bases, short reads and low complexity reads resulted in 382 720 reads that were analysed in this study. Approximately 0.4% (1639/382 720) of the analysed reads mapped to a viral sequence in the NCBI NT/NR database. The reads came from pool 2. Overall, based on the set thresholds, a total of 39 microbes were detected in the ticks. Bacteria were the most abundant microbes at 82% (32/39) followed by fungi at 15.4% (6/39) and viruses making up 2.6% (1/39).

### Detection of a tick phlebovirus

The number of reads per million sequenced in pool 2 aligning to phleboviruses in the NCBI NT/NR database was 1639 while the total number of reads across all assembled phlebovirus contigs (Contigs r) was 1055. The reads generated 3 contigs with 2 linked to phleboviruses, the largest contig (6485 bp) aligned to the L segment and one (665 bp) to the S segment while the third (526 bp) did not yield significant similarities after BLAST search in the NCBI database. The presence of the M segment could not be determined. Using the reference genome (GenBank accession # KU375576.1) selected by the pipeline, the percentage of the L segment of the reference genome covered by atleast one read or contig (Coverage breadth) was almost 100% with a coverage depth of 18.5×. The metrics for the nucleoprotein of the S segment were not provided by the pipeline.

### Analysis of the L and S gene sequences of the phlebovirus sequence

Analysis of a portion (Genbank accession # OR996374.1) of the 6485 bp L segment encoding the RdRp protein showed that RdRp spanned nucleotide positions 23–6485. Meanwhile, the NP sequence (Genbank accession # PP986954.1) within the 665 bp S segment was found on positions 17–665. A CD-search revealed that the RdRp protein sequence contained a conserved RdRp domain, typical of viruses in the Phenuiviridae family within the Bunyavirales order. This domain was situated between nucleotides 1874 and 3964, aligning closely with previously reported phleboviruses (Table 2). Additionally, the conserved N-terminus of the sequence featured a conserved endonuclease domain between nucleotides 218 and 457.

**Table 2.** Location of the conserved RdRp and endonucleases domains on the L segment of the query sequence (bold) against reference sequences

Virus accession No.	Nucleotide position of conserved protein sequences on the L segment of selected full length phleboviruses	
	RdPp	NP
NC_005214.1	1825–3915	178–396
NC_027140.1	1810–3897	157–399
NC_078070.1	1813–3903	157–399
KF892046.1	1846–3924	184–432
ON872644.1	1846–3924	184–426
**OR996374.1**	**1874–3964**	**218–457**
MN069028.1	1837–3927	232–420
MN025508.1	1837–3942	196–432
ON812282.1	1858–3942	196–435

### BLAST search and phylogenetic analysis of the viral sequences

**Figure 1.**
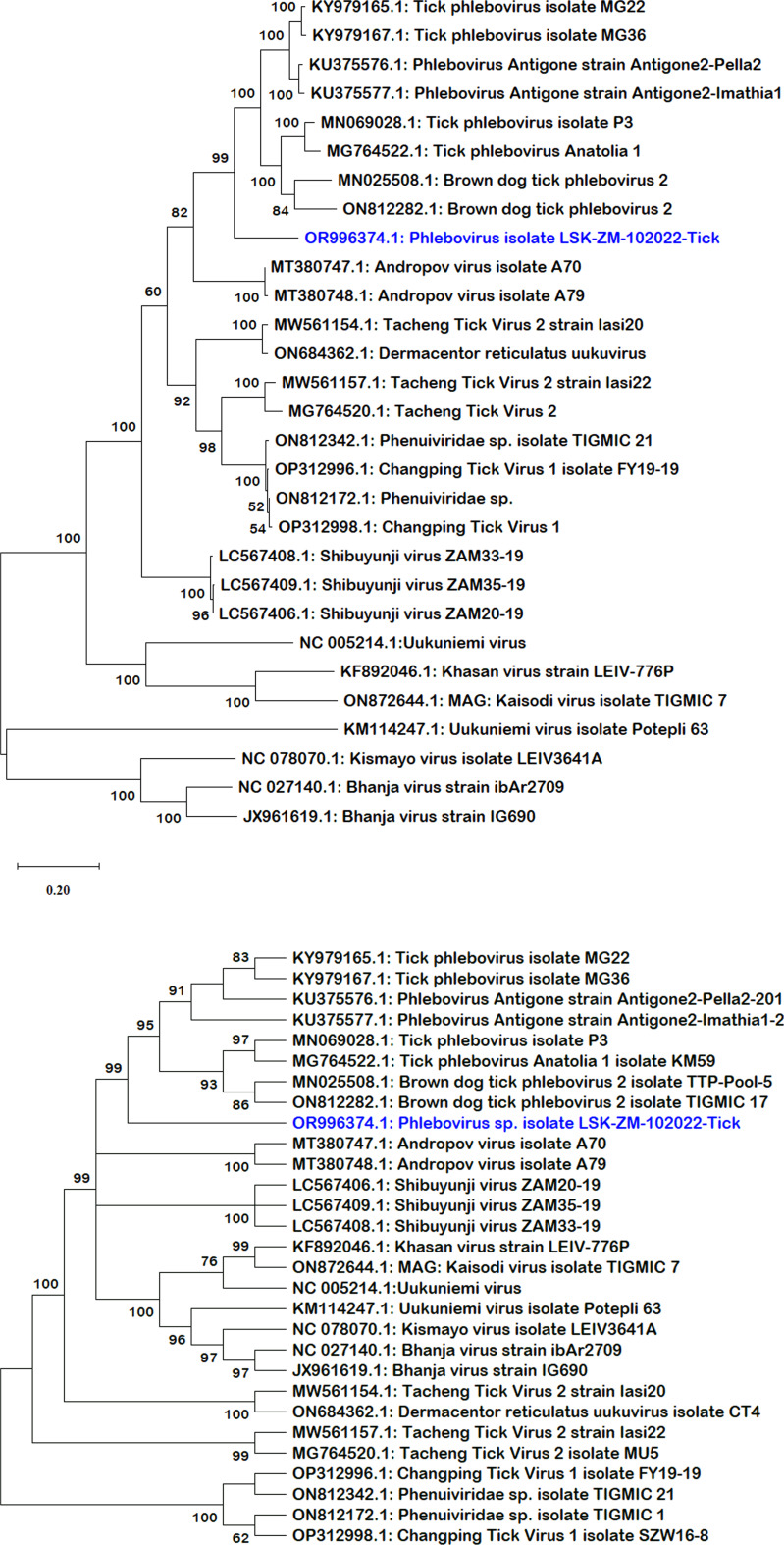
(a) Phylogenetic tree inferred based on partial RdRp gene sequences of the L segment, showing evolutionary relationships of phlebovirus isolate LSK-ZM-102022(blue) from Zambia and reference sequences. The analysis involved 29 nucleotide sequences. There were a total of 6766 positions in the final dataset. The reference sequences from GenBank are represented by accession numbers and strain names. Bootstrap values ≥60% are shown at branch nodes. The scale indicates the number of substitutions per site. (b) Phylogenetic analysis of phlebovirus isolate LSK-ZM-102022(blue) RdPp protein based on the amino acid sequences compared to reference sequences. The reference sequences from GenBank are represented by accession numbers and strain names. Bootstrap values ≥60% are shown at branch nodes. This analysis involved 29 amino acid sequences. There were a total of 515 positions in the final dataset.

**Figure 2.**
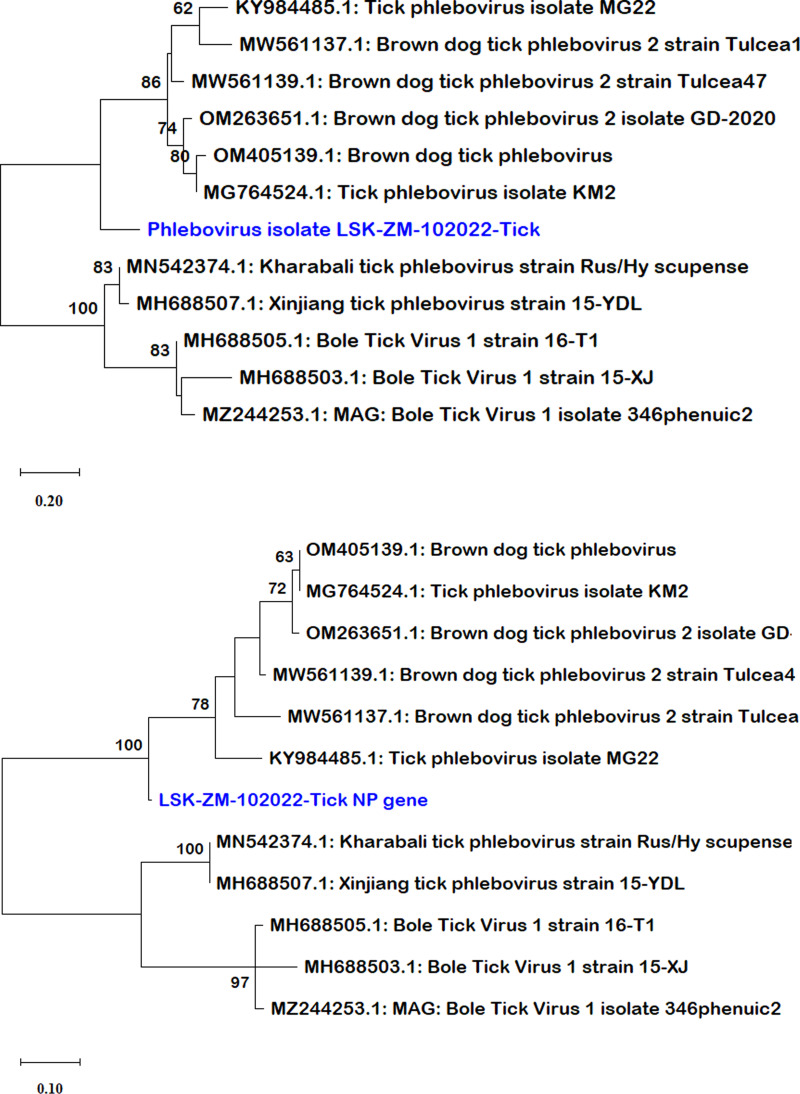
(a) Phylogenetic tree, inferred based on partial NP gene sequences of the S segment, showing evolutionary relationships of phlebovirus isolate LSK-ZM-102022 (blue) from Zambia and reference sequences. The analysis involved 12 nucleotide sequences. There were a total of 125 positions in the final dataset. The reference sequences from GenBank are represented by accession numbers and strain names. Bootstrap values ≥60% are shown at branch nodes. The scale indicates the number of substitutions per site. (b) Phylogenetic analysis of Phlebovirus isolate LSK-ZM-102022(blue) NP protein based on the amino acid sequences compared to reference sequences. The reference sequences from GenBank are represented by accession numbers and strain names. Bootstrap values ≥60% are shown at branch nodes. This analysis involved 12 amino acid sequences. There were a total of 90 positions in the final dataset. The scale indicates the number of substitutions per site.

BLAST search report of the segment L RdRp gene sequence of LSK-ZM-102022 indicated 74.92% nucleotide similarity to phlebovirus isolate MG31 (GenBank: KY979166.1) detected in *Rhipicephalus bursa* ticks from Turkey. Another BLAST search using the S segment NP gene sequence indicated 72% nucleotide similarity to a phlebovirus (Genbank: OM405139.1) reported in *Rhipicephalus* sp in Romania. The M segment was not among the sequences that mNGS was able to generate. Phylogenetically, the RdRp (Fig. 1a and b) and NP (Fig. 2a and b) nucleotide and amino acid sequences of LSK-ZM-102022 showed that the virus had a distinct novel lineage in a basal relationship to a cluster of related tick phleboviruses such as brown dog tick phlebovirus, phlebovirus antigone, tick phlebovirus Anatolia, and other tick phleboviruses. On both the RdRp and NP trees, LSK-ZM-102022shared a common ancestor with this cluster of tick phleboviruses

### Nucleotide sequence comparison among phleboviruses

Analysis of RdRp and NP pair-wise distances revealed notable positional differences with reference sequences (Table 3). RdRp nucleotide differences ranged from 19.5% to 32.6%, and amino acids from 9.3% to 35.8%, respectively. In contrast, the NP nucleotide sequences exhibited greater divergence, ranging from 30.4% to 47.1%, with amino acids differences ranging from 16.2% to 52.6%. Lower values indicated shared substantial amount of genetic material. The grouping based on pair-wise genetic distances mirrored the phylogeny-based grouping (Fig. 1a, b, 2a and b). Overall, the LSK-ZM-102022 phlebovirus sequence was genetically unique based on nucleotide and amino acid distances. Its closest relatives were tick phleboviruses from Turkey (Genbank accession #: MG764524.1, MG764522.1, KY984485.1, MN069028.1, KY979167.1 and KY979165.1) and Greece (Genbank accession #: KU375577.1, KU375576.1). Others were brown dog tick phleboviruses isolated from ticks in Romania (Genbank accession #: MW561139.1, MW561137.1, OM405139.1), China (Genbank accession #: ON812282.1), and Trinidad and Tobago (Genbank accession #: MN025508.1). The lowest pair-wise distance of 9.3%, based on the RdRp amino acid sequences, was observed between LSK-ZM-102022 and phlebovirus Antigone sequences (Genbank accession #: KU375577.1, KU375576.1) from Greece. This similarity was also noted between these sequences, but when comparing them using the RdRp nucleotide sequences, the distance was 19.5%.

**Table 3.** Comparisons of amino acid and nucleotide sequence pair-wise distances between LSK-ZM-102022 and reference sequences. Lowest values are in bold

Reference sequence	LSK-ZM-102022 NP gene (S segment)	
	Amino acids	Nucleotides
OM405139.1	0.2085	0.3498
OM263651.1	0.2231	0.3778
MW561139.1	**0**.**1625**	0.3041
MW561137.1	0.2231	0.3131
MG764524.1	0.2113	**0**.**2787**
KY984485.1	**0**.**1918**	0.3181
MN542374.1	0.4296	0.4071
MH688507.1	0.4296	0.4161
MH688503.1	0.4733	0.4441
MH688505.1	0.5205	0.4612
MZ244253.1	0.5261	0.4715
	RdRp gene (L segment)	
	Amino acids	Nucleotides
KU375577.1	**0**.**0930**	**0**.**1973**
KU375576.1	**0**.**0930**	**0**.**1948**
MG764522.1	0.1634	0.2534
ON812282.1	0.1791	0.2546
MN025508.1	0.1762	0.2541
MN069028.1	0.1814	0.2543
KY979167.1	0.1759	0.2456
KY179165.1	0.1756	0.2457
LC567408.1	0.2908	0.3097
MG764520.1	0.2465	0.2757
ON684362.1	0.2940	0.3116
MW561154.1	0.3577	0.3264
0P312998.1	0.2308	0.2804
MT380748.1	0.1822	0.2621
0N812342.1	0.2644	0.2679
MW561157	0.2882	0.3002
MT380747.1	0.1822	0.2546
0P312996.1	0.2989	0.3209
0N812172.1	0.2778	0.2766
LC567408.1	0.2908	0.3121

## Discussion

The recent emergence of severe fever with thrombocytopenia syndrome virus (SFTSV) and Heartland virus (HRTV) among humans underscores the urgent public health concern posed by phleboviruses (Li *et al*., 2016; McMullan Laura et al., 2012). These pathogens demand immediate attention and proactive measures to safeguard our communities from their potential impact. In Zambia, Shibuyunji virus detected in *Rhipicephalus* sp ticks of unknown species, remains the only known phlebovirus in the country (Simulundu *et al*., 2021). Addressing this gap is crucial for advancing knowledge and enhancing strategies to mitigate potential public health risks associated with phleboviruses, particularly TBPVs. In the present study, a potentially novel TBPV in *R. simus* ticks was found to occupy a distinct and lone evolutionary lineage among TBPVs.

The TBPV sequence was an incidental discovery among 39 sequences aligning to microorganisms in 10 tick samples collected during the search for mammarenaviruses in rodents (Munjita *et al*., 2022). The small number of ticks included in the study was purely because their initial collection was incidental and a 2-week follow up to capture more ticks yielded nothing. The presence of lower numbers of ticks can be attributed to fewer livestock or wild-animal populations that may attract ticks to the area (Li *et al*., 2018; Benyedem *et al*., 2022; Kumar *et al*., 2022), particularly that most adult *Rhipicephalus* sp ticks are expected to be common from December to April (Pegram *et al*., 1986). This highlights the critical role of host populations in influencing tick abundance.

Ixodid ticks in the genus *Rhipicephalus* are closely related species (Walker *et al*., 2000; Horak *et al*., 2002; Walker and Bouattour, 2003; Xu *et al*., 2016; Nicholson *et al*., 2019). They are known to harbour several pathogens of livestock and human concern including phleboviruses (Beati *et al*., 1995; Xia *et al*., 2015; Pereira *et al*., 2017; Nicholson *et al*., 2019; Rojas-Jaimes *et al*., 2021). However, as far as reviewed literature suggests, these findings present the first report of a TBPV in *R. simus* ticks. The findings extend the species level host range of TBPVs among ticks in the genus *Rhipicephalus* even though their epidemiology in ticks, animals, and humans in sub-Saharan Africa remains understudied (Kobayashi *et al*., 2017; Simulundu *et al*., 2021; Amoa-Bosompem *et al*., 2022; Ogola *et al*., 2023). Meanwhile, the discovery of any pathogen in *R. simus* is always of public health significance due to the ticks' aggressive abilities to infest and feed on humans (Horak *et al*., 2002). This unique characteristic of *R. simus* makes it a powerful agent for transmitting both novel and known zoonotic vector-borne pathogens from wildlife to humans.

Analysis of the LSK-ZM-102022 phlebovirus sequence provided compelling insights into its genetic makeup and evolutionary relationships. Firstly, the study identified key genomic regions within the L segment, crucial for understanding the virus's molecular characteristics. The RdRp protein, encoded by a segment spanning nucleotide positions 23–6485, exhibited a conserved RdRp domain typical of Phenuiviridae family viruses, specifically aligning with known phleboviruses (ICTV, 2024). The domain's conservation from nucleotides 1874–3964 suggests functional similarities with other members of the Bunyavirales order, highlighting the virus's evolutionary lineage. The presence of a conserved endonuclease domain within the L segment is typical of phleboviruses too (ICTV, 2024).

Phylogenetic analysis placed the LSK-ZM-102022-Tick sequence in a basal relationship to a cluster of related tick phleboviruses (Papa *et al*., 2016; Dinçer *et al*., 2017; Bratuleanu *et al*., 2022), underscoring its evolutionary position within the Phenuiviridae family. Notably, pair-wise genetic distance calculations for RdRp and NP sequences highlighted moderate divergence, indicating potential adaptations and genetic drift within these genomic regions. This diversity underscores the virus's capacity for genetic variability, which may influence its ability to adapt to new hosts and environments. The closest genetic relatives of LSK-ZM-102022 phlebovirus are identified as phleboviruses from ticks in Turkey and Greece (Papa *et al*., 2016; Dinçer *et al*., 2017), emphasizing regional associations and evolutionary connections among tick-borne viruses. The findings provide critical insights into the virus's epidemiology and evolutionary dynamics, essential for anticipating and mitigating potential public health risks associated with emerging tick-borne pathogens.

Overall, the genetic uniqueness and evolutionary relationships elucidated in this study position LSK-ZM-102022 as a notable member of the phlebovirus family, distinct yet closely related to tick-associated phleboviruses worldwide (Papa *et al*., 2016; Dinçer *et al*., 2017; Bratuleanu *et al*., 2022). It was unfortunate that available RdPp sequences for Shibuyunji virus, only known phlebovirus in Zambia before the findings from the current study, were too short for any meaningful comparisons with LSK-ZM-102022. Further research into their ecology, transmission dynamics and pathogenic potential will be crucial for enhancing preparedness and response strategies against emerging infectious diseases linked to tick vectors.

## Conclusion

The identification of a potentially novel TBPV in *R. simus* ticks highlights a significant addition to understanding the ecology and epidemiology of phleboviruses. The discovery underscores the urgent need for proactive measures to address emerging pathogens, particularly in regions like Zambia where TBPVs have been scarcely investigated. The genetic distinctiveness of LSK-ZM-102022 within the Phenuiviridae family suggests unique evolutionary adaptations that warrant further investigation into its transmission dynamics and potential pathogenicity. Given the aggressive feeding behaviour of *R. simus* ticks and their potential to transmit pathogens to humans, understanding the ecology and epidemiology of TBPVs in these vectors is critical. By expanding the understanding of phlebovirus diversity and transmission dynamics, the impact of these evolving threats can be mitigated.

## Data Availability

The data from which the results were generated for the current study may be available from the corresponding author on reasonable request and on condition that access to the data is granted by the CZ management.
